# Interplay of Topological States on TI/TCI Interfaces

**DOI:** 10.3390/ma13204481

**Published:** 2020-10-10

**Authors:** Tatiana V. Menshchikova, Sergey V. Eremeev, Vladimir M. Kuznetsov, Evgueni V. Chulkov

**Affiliations:** 1 Laboratory of Nanostructured Surfaces and Coatings, Tomsk State University, 634050 Tomsk, Russia; kuznetsov@rec.tsu.ru; 2 Laboratory Surface Phenomena Physics, Institute of Strength Physics and Materials Science, Siberian Branch of Russian Academy of Sciences, 634055 Tomsk, Russia; eremeev@ispms.tsc.ru; 3 Laboratory of Electronic and Spin Structure of Nanosystems, Saint Petersburg State University, 198504 Saint Petersburg, Russia; evguenivladimirovich.tchoulkov@ehu.eus; 4 Donostia International Physics Center (DIPC), 20018 San Sebastián/Donostia, Spain

**Keywords:** topological insulator, topological crystalline insulator, interfaces, density functional theory calculations

## Abstract

Based on first-principles calculations, we study electronic structure of interfaces between a $Z_{2}$ topological insulator (TI) SnBi${}_{2}$Te${}_{4}$ and a topological crystalline insulator (TCI) SnTe. We consider two interface models characterized by the different atomic structure on the contact of the SnTe(111) and SnBi${}_{2}$Te${}_{4}$(0001) slabs: the model when two materials are connected without intermixing (abrupt type of interface) and the interface model predicted to be realized at epitaxial immersion growth on topological insulator substrates (smooth interface). We find that a strong potential gradient at the abrupt interface leads to the redistribution of the topological states deeper from the interface plane which prevents the annihilation of the $\bar{\Gamma }$ Dirac states, predicted earlier. In contrast, a smooth interface is characterized by minor charge transfer, which promotes the strong interplay between TI and TCI $\bar{\Gamma }$ Dirac cones leading to their complete annihilation.The $\bar{M}$ topologically protected Dirac state of SnTe(111) survives irrespective of the interface structure.

## 1. Introduction

In the last decade, the strong impact of topology on electronic structure of many materials and related properties such as novel spin textures, surface and edge Dirac states, magnetic properties in two- and three-dimensional topological insulators, spin and charge transport as well as exotic superconducting states have been actively discussed [1,2,3,4,5,6,7,8]. The first class of such materials belongs to the $Z_{2}$ topological insulator phase. This phase is characterized by emergence of a single Dirac surface state in an inverted bulk energy gap which is topologically protected by the time-reversal symmetry. Most of the known TIs are represented by binary or ternary van der Waals compounds possessing quintuple-layer (QL) or septuple-layer (SL) structure as well as by compounds composed of alternating QL and SL structural blocks [9]. It is worth being noted that, owing to weak van der Waals coupling between structural layers, the surface is free from dangling bond states. The second class of materials represents so-called topological crystalline insulators in which even the number of gapless surface states are protected by the crystal mirror symmetry [10]. One of the most studied examples of these systems is SnTe [11,12,13,14,15,16,17]. The nontrivial electronic band structure of SnTe arises from band inversion at the L points of the bulk Brillouin zone combined with the mirror symmetry in the rocksalt fcc crystal structure with respect to the (110) plane. Owing to the non-trivial topology of the bulk band structure, the gapless surface states should arise on (001), (111), and (110) surfaces, which preserve the mirror symmetry. The SnTe(111) surface has been predicted to support Dirac cones centered at the $\bar{\Gamma }$ and $\bar{M}$ points of 2D Brillouin zone [13]. However, the surface potential effect on atomically flat polar SnTe(111) destroys the topological states being weakly protected by the crystal symmetry [14].

One of the relevant areas of research is the design of hybrid materials containing the interfaces between TIs with other materials allowing for creating novel 2D electronic systems with unique characteristics. For instance, such interfaces as graphene/TI combine both TI nontrivial spin textures with high electron mobility that was supported by experimental observation of a large variety of remarkable (spin) transport phenomena [18,19]. Magnetic insulator/TI contact demonstrates the exchange splitting in the Dirac cone [20,21,22,23] allowing realization of various practically useful quantum phenomena, such as topological magnetoelectric effect [24] and the quantum anomalous Hall effect [25,26]. Additionally, an interplay of Dirac and Rashba fermions can also provide a starting point for next generation of spintronics devices based on Rashba–Dirac coupled systems [27].

At the same time, much less attention was paid to interfaces formed by different TI classes like $Z_{2}$ topological insulator and topological crystalline insulator (TCI). TCI-based interfaces can demonstrate intriguing phenomena due to possible coexisting different Dirac cones and significantly expand the striking properties of the respective components, or improve technically significant characteristics of each other [28,29]. As an example, in Ref. [28], the authors have been studying the interface between $Z_{2}$ TI Bi${}_{2}$Te${}_{3}$(0001) and TCI SnTe(111) by means of tight-binding calculations. It was shown that at the interface the $\bar{\Gamma }$ Dirac states of Bi${}_{2}$Te${}_{3}$ and SnTe annihilate owing to their identical spin chirality [30]. However, at the $\bar{M}$ point, the Dirac state survives owing to the topological protection from the mirror symmetry of SnTe. Such state resides at each time-reversal invariant momentum $\bar{M}$ and should result in a highly conducting channel at the TI/TCI interface.

However, it is well known that, when the tight-binding method is used, the surface/interface spectrum is characterized by the absence of any surface/interface charge density redistribution thus retrieving the bulk-boundary-correspondence [31]. In the case of interfaces with the polar SnTe(111) surface, such perturbations can play a crucial role in the formation of different types of surface states and strongly affect the electronic properties. Moreover, the crystal structure of the interface can also influence the electronic structure and, therefore, it can be used as a promising tool for obtaining tailor-made electronic properties. The simplest interface structure implies “gluing” two materials along the interface plane. However, such kind of interface (abrupt) is realized when the diffusion processes are suppressed. At the same time, such processes can play a crucial role in the interface formation and lead to the conceptually new type of interface (smooth) which was not considered for the TI/TCI interface earlier. An example of spontaneous formation of the smooth interface is formation of well-ordered hexagonal MnBi${}_{2}$Se${}_{4}$ SL [32] on top of Bi${}_{2}$Se${}_{3}$ TI due to immersion of the epitaxially deposited Mn and Se atoms into the surface quintuple layer of a TI [22]. Following this procedure, it is possible to grow relatively thick films sandwiched by the remnant layers of the TI structural block [33] that has been realized very recently [34]. In this context, the ideal ingredient for TI/SnTe smooth interface can be SnBi${}_{2}$Te${}_{4}$, which is naturally composed of SL blocks which include SnTe bilayer in the middle.

Here, we consider SnBi${}_{2}$Te${}_{4}$(0001)/SnTe(111) interfaces on the base of density functional theory calculations. We analyze two interface models constructed as “gluing” of the SnTe(111) and SnBi${}_{2}$Te${}_{4}$(0001) slabs (abrupt model) and smooth interface model, where SnTe(111) slab is embedded in the interfacial SL of SnBi${}_{2}$Te${}_{4}$. We demonstrate that, in case of the abrupt interface, a rather wide and deep interface potential well is formed. This interface potential leads, on the one hand, to the appearance of trivial interface states throughout the gap, and, on the other hand, to impelling the $\bar{\Gamma }$ topological states to place deeper from the interface plane, which allows for surviving them in contrast to earlier prediction. In the case of the smooth interface, the depth of the interface potential is small, and its width is limited to several atomic layers. As a result, the trivial interface states do not arise at the Fermi level and the Dirac state at the $\bar{\Gamma }$ point is not observed either. However, in both interface models, the Rashba split surface state resides at $\bar{\Gamma }$ below the SnBi${}_{2}$Te${}_{4}$ bulk conduction band.

## 2. Calculation Methods

Density functional theory (DFT) electronic structure calculations were carried out using the projector augmented wave method [35,36] implemented in VASP [37]. The exchange-correlation energy was treated using the generalized gradient approximation with the PBE exchange-correlation functional [38]. The Hamiltonian contained scalar relativistic corrections, and the spin-orbit coupling was taken into account by the second variation method [39].

The energy convergence criterion was set to 10${}^{-6}$ eV for all types of calculations. Break condition for the ionic relaxation loop defined by forces smaller than 10${}^{-5}$ eV/Å. Integrations over the Brillouin zone are performed with a Γ-centered k-point grid of 11 × 11 × 1. The plane wave energy cutoff was chosen to be 219 eV, and it was kept constant through all calculations.

To simulate the interfaces between SnTe and SnBi${}_{2}$Te${}_{4}$, a periodic in all three directions heterostructures (without vacuum region) were considered. The abrupt interface is formed as “gluing” of SnTe and SnBi${}_{2}$Te${}_{4}$ slabs are composed of 49 and 42 layers, respectively. In the case of smooth interface, the cell is composed of 49 layers of SnTe(111) and 39 layers of SnBi${}_{2}$Te${}_{4}$(0001). The interfacial interlayer spacings as well as the atomic positions within the first (closest to the interface) SL of SnBi${}_{2}$Te${}_{4}$ and ten near-interface atomic layers of SnTe were optimized, while the interatomic distances within the middle parts of both slabs were fixed. Interfacial relaxation was carried out by the DFT+D3 method that correctly describes the van der Waals interactions [40,41].

## 3. Results and Discussion

**Figure 1:**
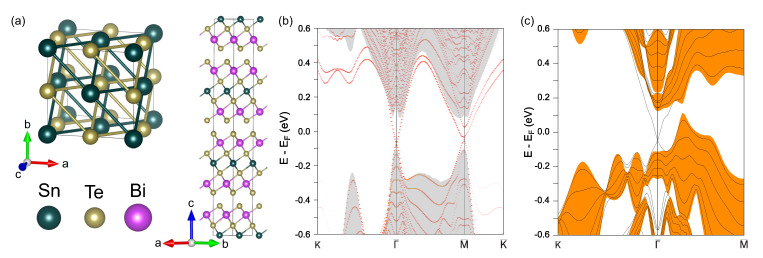
Crystal structure of bulk SnTe (left panel) and SnBi${}_{2}$Te${}_{4}$ (right panel) (**a**). Surface electronic structures of the nonpolar SnTe(111) (**b**) and SnBi${}_{2}$Te${}_{4}$(0001) (**c**). The grey (orange) background corresponds to the SnTe (SnBi${}_{2}$Te${}_{4}$) projected bulk band structure.

The relativistic band structure of SnTe is known from the end of 60th [42]. It is characterized by the bulk band inversion of the Sn and Te states at the L points of the bulk Brillouin zone of the rocksalt SnTe. Later, it was shown that this band inversion in combination with the mirror symmetry results in formation of topologically protected surface states in this material [11]. According to the angle-resolved photoemission spectroscopy (ARPES) data [43], the (111) surface of the cubic SnTe (Figure 1a, left panel) possesses the $\bar{\Gamma }$ and $\bar{M}$ point Dirac cones in the spectrum, as well as it demonstrates the absence of the trivial surface states at the Fermi level. However, it contradicts to the DFT calculation results for polar SnTe(111) surfaces, which revealed the presence of trivial spin-split states propagating over the entire two-dimensional Brillouin zone (BZ). These states arise from the surface potential effect, which also destroys weakly protected topological surface states [14]. Eradication of the trivial surface states as well as revealing of the Dirac states on this surface can be achieved by surface passivation [14] or by forming nonpolar conditions on the (111) surface, which most likely take place on a real surface in the experiment. To illustrate the second scenario and mimic the nonpolar conditions on the SnTe(111) surface, we constructed the $1\times \sqrt{3}$ supercell containing two atoms in each atomic layer with one surface atom removed, resulting in the reconstruction containing simultaneously anion and cation atoms on the surface. The spectrum of such nonpolar surface, unfolded onto the original 1 × 1 2D BZ [44,45], presented in Figure 1b, shows that surface electronic structure is characterized by the topological states located at the $\bar{\Gamma }$ and $\bar{M}$ points, in fine agreement with the experiment.

For the second ingredient of TCI/TI interface, we have chosen SnBi${}_{2}$Te${}_{4}$ $Z_{2}$ TI [9,46,47], which has reasonable matching with in-plane lattice parameters of the SnTe(111) film and, even more important, naturally includes SnTe bilayers. SnBi${}_{2}$Te${}_{4}$ is composed of hexagonally ordered SL blocks stacked along the *c* axis and separated by van der Waals spacings (Figure 1a, right panel). The SL building block can be obtained from the original QL block of binary Bi${}_{2}$Te${}_{3}$ by introducing the SnTe bilayer between the Bi atomic layer and the central Te one. As can be seen in Figure 1c, the surface electronic structure of SnBi${}_{2}$Te${}_{4}$ accommodates single Dirac cone at the $\bar{\Gamma }$ point and is free of trivial surface states. The Dirac state has positive (clockwise) spin helicity above the DP and negative helicity in the lower part of the cone [47] like the $\bar{\Gamma }$ Dirac state of SnTe(111) [14].

**Figure 2:**
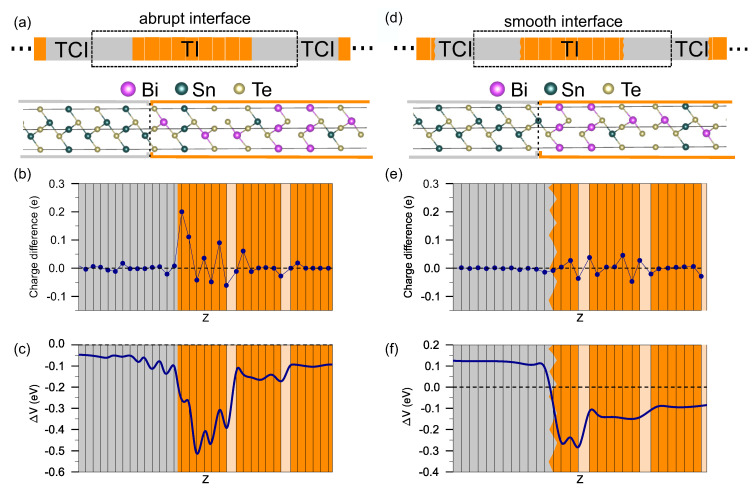
Schematic view (**top**) and atomic geometry (**bottom**) of the supercell containing abrupt TI/TCI interfaces (**a**). Vertical dashed line in bottom part of panel (**a**) shows a border between two materials SnTe and SnBi${}_{2}$Te${}_{4}$. Atomic charge difference with respect to the electronic charge in center of the SnBi${}_{2}$Te${}_{4}$ (orange) and SnTe (grey) slabs, respectively (**b**). Change of the total electrostatic potential averaged over xy planes with respect to that in the bulk of the SnBi${}_{2}$Te${}_{4}$ (orange) and SnTe (grey) slabs (**c**) for abrupt interface. Vertical black lines in the panels (**b**,**c**) mark the position of atomic layers and light orange regions show van der Waals spacing in SnBi${}_{2}$Te${}_{4}$. (**d**–**f**) is the same as (**a**–**c**) except for a smooth interface.

We consider two different interface structures: abrupt and smooth models, discussed above. The first one is formed as ”gluing” of SnTe and SnBi${}_{2}$Te${}_{4}$ slabs (Figure 2a). According to the total energy calculations, the Sn-terminated side of the SnTe slab energetically more favorable (230 meV) with respect to the Te-terminated side. For this reason, we use the Sn-terminated slab for construction of the interface. The lattice parameter of the rocksalt SnTe in the hexagonal (111) plane, 4.469 Å, only by ≈1.6 % exceeds the lattice parameter of SnBi${}_{2}$Te${}_{4}$ (4.397 Å). For construction of the interfaces, which requires a single lattice parameter of the cell in the interface plane, we use the parameter of TI. A small reduction of the parameter *a* provided that the unstressed state of the lattice is preserved, leads to the rhombohedral distortion of the cubic structure of SnTe. It is worth noting that the small distortion does not destroy the band inversion, which now take place in Z and and three L points of the distorted BZ and, consequently, the $\bar{\Gamma }$ and $\bar{M}$ Dirac states should remain in the SnTe(111) surface spectrum.

The strategy of the smooth interface construction is based on a recently proposed novel type of interface between a magnetic insulator and a topological insulator of the Bi${}_{2}$Se${}_{3}$ family [22,33,34]. It implies the immersion of the epitaxially deposited magnetic atoms into the surface quintuple layer of a Bi${}_{2}$Se${}_{3}$ substrate which results in formation of the magnetic film sandwiched by the remnant layers of this quintuple layer. Moreover, as was shown in Ref. [33], by inserting the number of bilayers in the “grown-in” film, one can obtain the sandwiched layer with thickness up to several nanometers. In our case, instead of magnetic insulator and Bi${}_{2}$Se${}_{3}$ TI, we use non-magnetic SnTe TCI and SnBi${}_{2}$Te${}_{4}$ TI, respectively. In order to find out that the described immersion process with magnetic material is possible for non-magnetic SnTe bilayers, we consider, similar to Ref. [33], the energy difference between two structures: the SnTe bilayers on top of SnBi${}_{2}$Te${}_{4}$ SL and in the middle of SL. Comparison of the total energies of these configurations clearly indicates that the immersed bilayer is much more energetically favorable as compared to the case of the bilayer standing on the surface (energy gain is ≈ 90 meV per cell consisting of nine atoms). With an increase in the number of SnTe bilayers, it is still advantageous for them to be inside the SL block, and the gain in energy remains approximately the same. Thus, the formation of the smooth interface is also possible for TI/TCI (Figure 2b).

To analyze the characteristics of these two types of interface, we implement the Bader charge analysis [48]. Figure 2b shows the difference between the Bader atomic charges in the SnTe/SnBi${}_{2}$Te${}_{4}$ structure with abrupt interface and those in the central, bulk-like, layers of SnTe and SnBi${}_{2}$Te${}_{4}$ slabs, respectively. As can be seen from the figure, the SnBi${}_{2}$Te${}_{4}$ SL adjacent to the interface plane demonstrates large charge redistribution and maximum charge transfer occurs on the outer Te layer. At the same time, the charge redistribution in the SnTe part of the structure is relatively small, like in case of the interface between TI and normal [49] (magnetic [20,50]) insulator. Such a change in the electronic charge density leads to substantial modification of the electrostatic potential at the TI/TCI interface. To catch such modification at the interface region, we consider the change of the potential with respect to that in the center of SnBi${}_{2}$Te${}_{4}$ and SnTe slabs, respectively. As can be seen from Figure 2c, the abrupt interface is characterized by strong potential gradient at the boundary between SnBi${}_{2}$Te${}_{4}$ (orange) and SnTe (grey) regions, which is expressed in a sharp and strong subsidence (∼500 meV) of the potential within the SL block of SnBi${}_{2}$Te${}_{4}$ adjacent to the interface plane, forming a deep potential well. On the SnTe side, a decline in the potential (involving six atomic layers) also takes place. In contrast, the potential change for the smooth interface is much less pronounced and spreading only over five atomic layers (Figure 2f) indicating negligible perturbation at the interface region. This behavior of the interface potential correlates with atomic charge differences at the smooth interface (Figure 2e) which are much smaller as compared with the abrupt interface structure.

**Figure 3:**
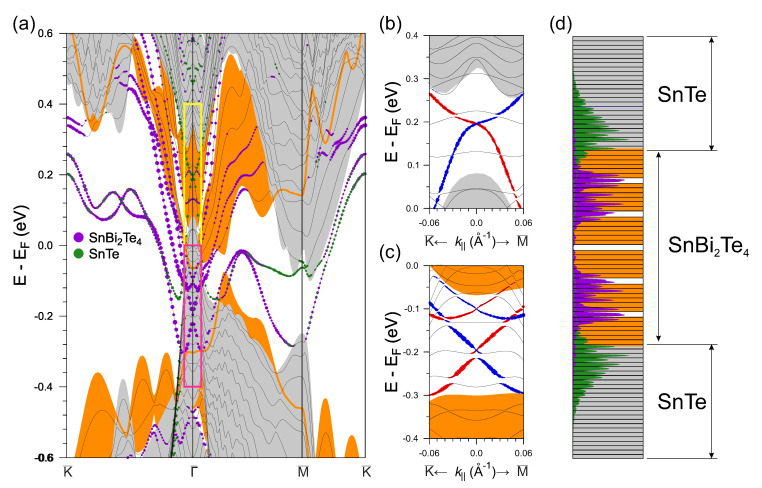
Electronic structure of the abrupt SnBi${}_{2}$Te${}_{4}$/SnTe interface (**a**). Orange (grey) background corresponds to SnBi${}_{2}$Te${}_{4}$ (SnTe) projected bulk band structure. The radii of the color circles reflect the localization of the states in the near-interface layers. Magnified view of the $\bar{\Gamma }$ Dirac states of SnTe (yellow rectangle in (**a**)) (**b**) and SnBi${}_{2}$Te${}_{4}$ (magenta rectangle in the panel (**a**)) (**c**), where size of red and blue circles reflects values of the positive and negative in-plane components of the spin eigenvalues, respectively (**b**,**c**). The charge density ρ(z) of the SnBi${}_{2}$Te${}_{4}$ (SnTe) Dirac states integrated over the xy plane (**d**).

Electronic structure of the abrupt interface is shown in Figure 3. As can be seen from the figure, the bulk band gaps of SnBi${}_{2}$Te${}_{4}$ and SnTe overlap at the $\bar{\Gamma }$ point with the bulk states of the counterpart subsystem. It should be noted that such overlap is realized for the interface structure with an in-plane lattice parameter $a=a_{\mathrm{SnTe}}$ too. Strong potential modification at the abrupt TI/TCI interface leads to emergence of the spin-split trivial surface states, like in cases of Bi${}_{2}$Se${}_{3}$/MnSe [20] and Bi${}_{2}$Se${}_{3}$/ZnSe [49] interfaces. The deep interface potential also results in a strong modification of the dispersion of the $\bar{M}$ Dirac state of SnTe and its upward energy shift towards the conduction band. It should be mentioned that the $\bar{M}$ state of SnTe(111) penetrates deeply into the bulk [14] and hence its convergence is sensitive to the slab thickness. In our calculation where SnTe slab consists of 49 atomic layers, only the $\bar{M}$ state has a small gap.

A striking difference between our results and those presented in Ref. [28] predicting the annihilation of the $\bar{\Gamma }$ Dirac states for the Bi${}_{2}$Te${}_{3}$/SnTe abrupt TI/TCI interface is a presence of two spin-polarized topological surface states at the $\bar{\Gamma }$ point. The first one resides in the gap of the bulk states of SnTe at ≈0.2 eV (Figure 3b) and the second Dirac state is located at −0.2 eV in the bulk gap of SnBi${}_{2}$Te${}_{4}$ states along with the Rashba-split trivial state (Figure 3c). The main reason for the difference with earlier tight-binding calculations, which do not take the interface potential into account, is the presence of the potential well at the interface in the DFT calculation. It was shown earlier [20,49] that the interface potential in the structures containing layered TI is responsible for the emergence of the trivial trapped interface states. In addition, it leads to a spatial shift of the topologically protected Dirac state deep into the TI film. In the abrupt TI/TCI case, the strong interface potential gradient also causes the shift of the Dirac state of SnBi${}_{2}$Te${}_{4}$ from the interface plane towards the second SL (Figure 3d). At the same time, the Dirac state of SnTe is mostly localized in the SnTe region only slightly penetrating into the outer SL of SnBi${}_{2}$Te${}_{4}$. As a result, these two Dirac states almost do not overlap in the real space that prevents their annihilation.

**Figure 4:**
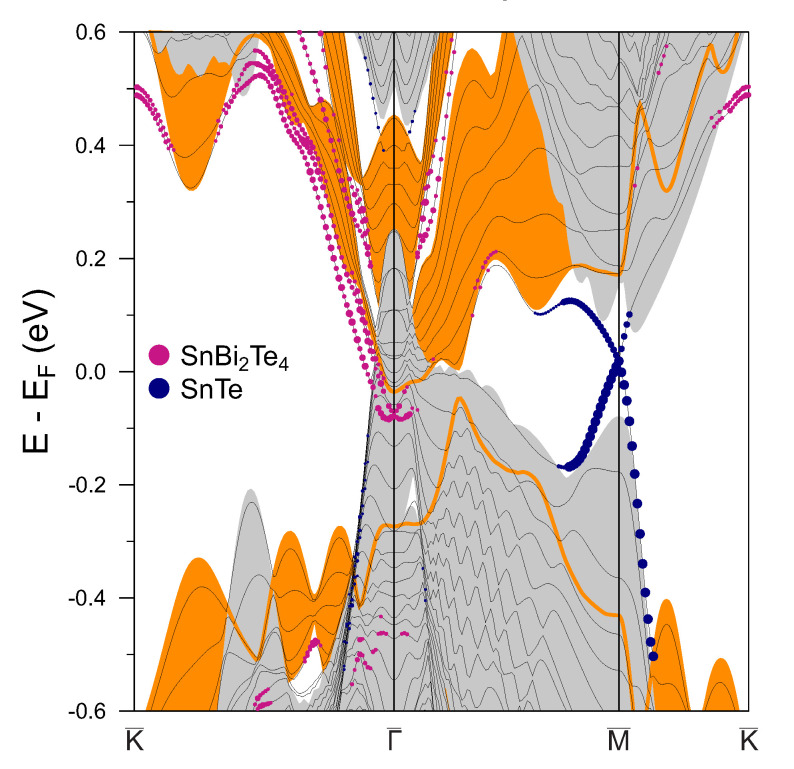
Electronic structure of the smooth SnBi${}_{2}$Te${}_{4}$/SnTe interface. Orange (grey) background corresponds to SnBi${}_{2}$Te${}_{4}$ (SnTe) projected bulk band structure. The radii of the purple (navy blue) circles reflect the localization of the states in the near-interface SnBi${}_{2}$Te${}_{4}$ (SnTe) layers.

In case of the smooth interface, the electronic structure qualitatively changes with respect to the abrupt interface structure. As can be seen from the Figure 4, bulk band gaps of interface components (SnBi${}_{2}$Te${}_{4}$ and SnTe) also overlap with bulk states of the counterpart at the $\bar{\Gamma }$ point. Negligible perturbations in the interface potential for the smooth interface structure induce only small splitting off the interface state from the SnBi${}_{2}$Te${}_{4}$ bulk conduction band in close vicinity to the $\bar{\Gamma }$ point, which, like in an abrupt interface case, acquires a Rashba spin splitting. Along with this, we found complete annihilation of the Dirac states at the $\bar{\Gamma }$ point, which is caused by a strong interplay between two Dirac cones [30] belonging to the SnTe and SnBi${}_{2}$Te${}_{4}$ slabs similar to the results of Ref. [28]. Such interplay is related with the absence of the topological states relocalization which comes from the minor charge transfer at the smooth interface and, as a consequence, from negligible perturbation in the interface potential. The lack of a deep potential well at the smooth interface also results in undisturbed linear dispersion of the $\bar{M}$ Dirac state residing in the SnTe bulk band gap.

## 4. Summary and Conclusions

In summary, we have studied the electronic structure of two structural types of Ti/TCI interface. The first interface is organized as contact of the Sn-terminated side of the SnTe(111) slab and SnBi${}_{2}$Te${}_{4}$(0001) (abrupt type). The second one is the smooth interface predicted to be realized at epitaxial immersion growth on topological insulator substrates. Despite the smooth interface formation being energetically favorable, the abrupt interface can be realized under certain conditions, in particular, when interdiffusion is suppressed. We have shown that the interplay of topological states belonging to the TI and TCI slabs can strongly depend on the interface atomic structure. We revealed that the strong potential gradient at the abrupt interface prevents the annihilation of TCI’s and TI’s Dirac cones at the $\bar{\Gamma }$ point, predicted in the earlier model; however, it also gives rise to the emergence of the trivial interface states throughout the gap. In the case of the smooth interface, characterized by negligible interface potential, there are no trivial interface states in the spectrum and the formation of the Dirac state at the $\bar{\Gamma }$ point is not observed.

## Abbreviations

The following abbreviations are used in this manuscript:

TI	Topological insulator
TCI	Topological crystalline insulator
QL	Quintuple-layer
SL	Septuple-layer
DFT	Density functional theory
VASP	Vienna Ab-initio Simulation Package
PBE	Perdew–Burke–Ernzerhof
ARPES	According to the angle-resolved photoemission spectroscopy
BZ	Brillouin zone
